# Optimizing recruitment to a prostate cancer surveillance program among male BRCA1 mutation carriers: invitation by mail or by telephone

**DOI:** 10.1186/1897-4287-11-17

**Published:** 2013-12-10

**Authors:** Anna Galor, Cezary Cybulski, Jan Lubiński, Steven A Narod, Jacek Gronwald

**Affiliations:** 1 Department of Genetics and Pathology, International Hereditary Cancer Center, Pomeranian Medical University, Połabska 4, 70-115 Szczecin, Poland; 2 The Centre for Research in Women’s Health, University of Toronto, Ont, Canada

## Abstract

The effectiveness of a genetics-based public health screening programs depend on the successful recruitment of subjects who qualify for intensified screening by virtue of a positive genetic test. Herein we compare the effectiveness of a mailed invitation and follow-up phone call for non-responding subjects and an initial invitation by telephone addressed to male BRCA1 mutation carriers for prostate screening.

The final participation rate was 75% (42 of 56) for men who were initially contacted by mail (and follow-up phone call) and 81% (30 of 37) for men who were initially contacted by telephone. Among the men who were initially contacted by mail, it was necessary to telephone 54% of these patients (30 of 56).

After a calculation of the cost-effectiveness related to these results, we conclude that if the costs of the phone call were to exceed the costs of the letter by 2.5 times or more, then savings would be arranged by initiating contact with a mailed invitation.

## Introduction

Mutations in BRCA1 and BRCA2 confer high risks of breast and ovarian cancer in women [1-4]. Men who carry a mutation in these genes are at increased risk of prostate cancer [5-7] Extensive population screenings for prostate cancer are of limited cost-effectiveness, however if they are addressed to high risk groups of patients only, the cost-effectiveness seem to be satisfactory, and BRCA1 and BRCA2 carriers may benefit from cancer surveillance using PSA and rectal examination. The success of a genetic-based public health screening program depends on the efficient recruitment of subjects who qualify for intensified screening by virtue of a positive genetic test. There are several methods by which healthcare professionals might encourage patients to participate in enhanced screening programs. The passive approach depends on public awareness the potential participant is made aware of (that screening is available) and approaches the screening center on their own volition or upon the recommendation of their physician; in this case, recruitment is enhanced by media coverage, as well as by advertisement [8]. Two active recruitment methods include mailing a letter of invitation to the subject or a phone call from a trained staff member [9,10]. It has been shown that the mailed invitation approach is less expensive than direct active recruitment (telephone call), however it may be less effective [11,12] and may not be cost-effective in terms of the actual number of subjects recruited successfully. In some jurisdictions, recruiters will initially use a mailed approach and follow this up with a phone call to those subjects who did not respond to the initial mailed request. An alternative for mail invitation would be an email invitation, however nowadays it is of limited use if the program is directed towards older patients, who do not use the internet frequently, or inhabitants of rural areas with not satisfactory access to internet [13].

To our knowledge, there are no studies which evaluate the effectiveness of various recruitment methods for prostate screening among male BRCA1 mutation carriers. In this study, we compare the effectiveness of two approaches: 1) a mailed invitation and follow-up telephone call for patients who did not respond and 2) an initial invitation by telephone.

## Materials and methods

The Study was performed between November 2008 and March 2009 in the Outpatient Clinic of Genetic Oncology of Pomeranian Medical University, Szczecin, Poland. Men aged 40–69, who were residents of the Western Pomeranian district and who were known to carry a BRCA1 mutation were eligible to take part in the Polish Ministry of Science project (PBZ-MNiSW-05/I/2007/02) on evaluation of the effectiveness of population-based screening of breast, colon and prostate cancer through the use of DNA testing for the detection of an increased genetic predisposition to these cancers [13]. Within this project one of the subject groups were men, BRCA1 mutation carriers who show increased risk of prostate cancer. Screening for prostate cancer was offered within the program and included measurement of serum PSA (prostate specific antigen) and an urological prostate examination. Preliminary results of this study have been published elsewhere [14]. All patients signed an informed consent for inclusion in the study. The study was approved by the Ethic Committee of Pomeranian Medical University.

Men with a known BRCA1 mutation, who had been previously tested in the genetic clinic were eligible for the study. 99 male BRCA1 mutation carriers were identified from the records of the Department of Genetics and Pathology of PUM. Of these, 93 men were alive and were eligible to participate in the study. The following methods were used in order to encourage patients to participate in program:

a) A mailed invitation was sent to 56 BRCA1 carriers (chosen at random from the 93). The mailed invitation included a short description of the study, the prostate examination, the significance and possible benefit of the examination. The letter included a proposed time and date of appointment. The invitation also indicted that changes could be arranged if the proposed time was inconvenient. In the event that the subject did not respond to the mailed invitation, a follow-up phone call was made. In this case, the study nurse invited the patient to the program and explained the significance of examinations, possible benefits and was available to answer questions. If the subject wished to participate, an appointment was made.

b) The remaining 37 BRCA1 carriers were approached directly by a participating study nurse by telephone. The nurse invited the patient to the program and explained the significance of examinations, possible benefits and also answered questions. If the subject wished to participate, an appointment was made.

## Results

Among the 37 patients selected to be invited by an initial phone call, all were contacted and 30 (81%) agreed to participate. Three men refused and four men deferred participation. Among the 56 subjects who received a letter of invitation, 26 (46%) agreed to participate and 30 did not respond. A nurse attempted to contact each of these 30 non-responding subjects but was unable to contact six of them. Of the 24 men who were contacted successfully, 16 agreed to participate, 3 refused and 5 deferred participation.

**Figure 1:**
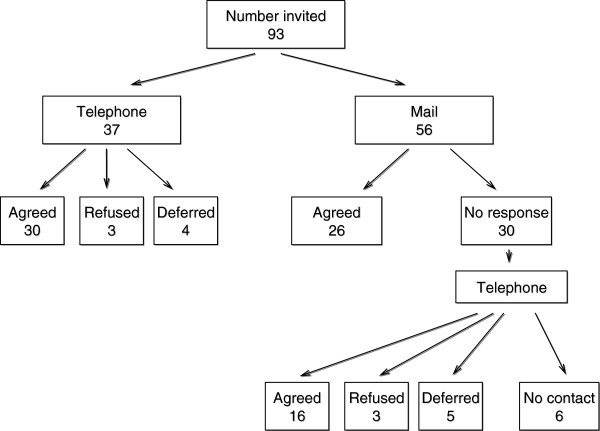
Flowchart of patient participation.

In summary, the ultimate participation rate was 81% (30 of 37) for men who were initially contacted by telephone and 75% (42 of 56) for men who were initially contacted by mail. Among the men who were contacted initially by mail, it was necessary to telephone 54% of these patients (30 of 56). Flowchart of patient participation is shown on Figure 1.

## Discussion

In this study, our overall response rate was high (77%) indicating a strong interest in prostate cancer screening among Polish men with a BRCA1 mutation. The response rate is high as compared to other studies which analyzed the effectiveness of invitations performed by the same means [15,16]. In general, men are less likely than woman to take part in screening programs [17]. However, in this study all of the participants were carriers of a BRCA1 mutation and had previously made the decision to undergo genetic counseling and genetic testing.

Our findings indicate a small advantage of initial phone call invitations over those made by mail. In the case of phone calls, 81% of the invited participants ultimately reported to the study center, whereas the proportion was 75% for mail invitations followed by a telephone call for non-responders. The majority of men who were initially approached by mail also required a telephone call eventually and we were only able to reduce the number of telephone calls by 46% by sending an initial mailed invitation.

Rezner et al. showed that recruitment performed by direct phone calls as compared to letter invitations may improve recruitment effectiveness from 9% to 52% [18]. Wong et al. and others studies found that a combination of several invitation methods can increase uptake, especially, when mail invitation is followed by a phone invitation [19-21]. Invitations by telephone are more personal and may be more understandable to patients. Furthermore, the time and date of the visit can be adjusted immediately. The study determined that even for groups of patients who initially ignored a letter invitation for a screening program, it is possible to significantly increase the participation rate after telephone calls.

Our study did not directly compare the cost-effectiveness of the two methods. For phone call invitations, costs include the time spent by staff on performing calls, as well as the telephone call rates. For mail invitations, the calculation should include the cost of mail materials, postal rates and the time spent by staff on preparing invitations. In this study, using the phone call only approach, it required a mean of 1.23 phone calls to obtain one study subject. In the approach with mail first then phone call it required a mean of 1.33 letters and 0.71 phone calls per study subject recruited. If the costs of the letters and phone calls were equal, then the phone call first method would be more cost-effective. However, if the cost of the phone call were to exceed the cost of the letter by 2.5 times or more, then savings would be realized by initiating contact with a mailed invitation.

An alternative to mail invitation is email invitation. The use of email invitation may significantly reduce the costs. Email correspondence is much faster and may include additional tools, like function indicating the receipt of a message, what would be of value for program management. This type of invitation is more interactive and asking possible questions as well as answering is fast and easy. However, in our study we did not evaluate this method since the program was directed to significant proportion of older man, who generally do not use email, and were frequently coming from rural areas with limited access to the internet. However, we think that in the near future email invitation method will not only become an alternative for mail invitations but that it might replace it.

## Competing interests

Authors declare that they have no competing interests.

## Authors' contributions

Conception and design: AG; drafting the manuscript: AG; critical revision for important intelectual content: JG, CC; final approval: JL, JG, CC, SA N, AG. All authors read and approved the final manuscript.
